# Immunotherapy innovations in triple-negative breast cancer: targeting checkpoints, combinations, and biomarkers

**DOI:** 10.3389/or.2026.1802090

**Published:** 2026-04-22

**Authors:** Zi-Xin Wang, Ju-Hang Chu, Ya-Ru Wang, Lu-Yao Huang, Ming-Ping Qian

**Affiliations:** 1 Department of General Surgery, Shanghai Tenth People’s Hospital, School of Medicine, Tongji University, Shanghai, China; 2 Yinshan Lake Hospital of Suzhou Wuzhong District, Jiangsu, China; 3 Department of General Surgery, Shanghai Tenth People’s Hospital Chongming Branch, Shanghai, China

**Keywords:** biomarker-driven therapy, combination therapy, immune checkpoint inhibitors, immunotherapy, triple-negative breast cancer

## Abstract

Triple-negative breast cancer (TNBC), an aggressive subtype lacking estrogen receptor (ER), progesterone receptor (PR), and HER2 expression, accounts for 10–20% of breast cancers and is characterized by high metastatic potential and poor survival outcomes. Despite advancements in chemotherapy, the 5-year survival rate for metastatic TNBC remains below 30%, underscoring the need for innovative therapeutic approaches. This review comprehensively examines recent breakthroughs in TNBC immunotherapy, focusing on immune checkpoint inhibitors (ICIs), combination strategies, and biomarker-driven therapy. Landmark trials such as KEYNOTE-355 and IMpassion130 have demonstrated that combining PD-1/PD-L1 inhibitors with chemotherapy improves survival in PD-L1-positive metastatic TNBC. Beyond monotherapy, combination therapies—including dual checkpoint inhibition, PARP inhibitors in BRCA-mutated tumors, and antibody-drug conjugates (ADCs) —show promise in overcoming resistance and enhancing antitumor immunity. Emerging targets further expand therapeutic possibilities, though their paradoxical roles as biomarkers and immunosuppressive mediators require precision-based approaches. Biomarkers like PD-L1, tumor-infiltrating lymphocytes (TILs), tumor mutational burden (TMB), and circulating tumor DNA (ctDNA) are critical for patient stratification and predicting immunotherapy response. Despite progress, challenges persist, including tumor heterogeneity, resistance mechanisms, and access to advanced therapies. Future directions emphasize next-generation ICIs, optimized combination regimens, and AI-driven biomarker integration to achieve durable, personalized treatments. This review underscores the potential of immunotherapy to redefine TNBC management while highlighting the imperative for continued innovation to address unmet clinical needs.

## 1 Introduction

Triple-negative breast cancer (TNBC), defined by the absence of estrogen receptor (ER), progesterone receptor (PR), and HER2 expression, represents 10–20% of all breast cancers and is associated with aggressive tumor biology, early metastasis, and poor prognosis compared to other subtypes (1). TNBC disproportionately affects younger women and individuals of African ancestry. Therapeutic options remain limited to cytotoxic chemotherapy due to the absence of actionable hormonal or HER2-targeted receptors (2, 3). Despite advancements in systemic therapy, accelerated disease progression, treatment resistance, and a 5-year survival rate below 30% for metastatic cases highlight the critical demand for novel therapeutic strategies (4, 5).

The emergence of immunotherapy has revolutionized TNBC management, leveraging its unique immunogenic features such as higher tumor mutational burden, PD-L1 expression, and tumor-infiltrating lymphocytes (TILs) (2, 6). Immune checkpoint inhibitors (ICIs), particularly PD-1/PD-L1 inhibitors such as Pembrolizumab and Atezolizumab, have demonstrated clinically meaningful efficacy in PD-L1-positive patients, with landmark trials such as IMpassion130 reporting a 10.5-month overall survival benefit (7, 8). Beyond ICIs, emerging approaches including antibody-drug conjugates (ADCs), PARP inhibitors, and biomarker-driven combinations, are transforming the therapeutic landscape, providing opportunities for personalized and durable responses (9, 10).

While several comprehensive reviews have summarized the landscape of TNBC immunotherapy, this review provides a unique conceptual advancement by critically dissecting the paradoxical roles of emerging targets like LAG-3, which serve as both prognostic biomarkers and immunosuppressive mediators. Furthermore, we move beyond cataloging combination strategies to analyze the synergistic mechanisms underpinning different combination platforms—chemotherapy, PARP inhibitors, and ADCs—thereby offering a framework for rational regimen design. By integrating the latest evidence on biomarker heterogeneity and resistance mechanisms, this review aims to chart a course toward a more precise and durable personalized immunotherapy paradigm for TNBC.

## 2 Immune checkpoint inhibitors in TNBC

ICIs function by disrupting inhibitory signaling pathways that suppress T-cell activation, thereby restoring antitumor immune responses. In TNBC, this mechanism has translated into clinically meaningful benefits, particularly in PD-L1-positive subsets, where PD-1/PD-L1 inhibitors combined with chemotherapy have improved survival outcomes (11). Beyond their direct immunomodulatory effects, ICIs enhance the immunogenicity of chemotherapy-induced tumor cell death by promoting neoantigen release and dendritic cell activation. The following sections explore these next-generation inhibitors and their potential to redefine TNBC immunotherapy through mechanistic synergy and precision targeting.

### 2.1 PD-1/PD-L1 inhibitors

The programmed cell death protein 1 (PD-1) and its ligand PD-L1 play a critical role in tumor immune evasion by negatively regulating T-cell activation. PD-1/PD-L1 interaction suppresses effector T-cell function, promotes regulatory T-cell (Treg) immunosuppressive activity, and maintains an immunosuppressive tumor microenvironment, enabling cancer cells to escape immune surveillance (12). In TNBC, targeting PD-1 or PD-L1 with monoclonal antibodies has shown significant promise, particularly in PD-L1-positive patients.

Pembrolizumab, a PD-1 inhibitor, has significantly advanced the treatment of TNBC, particularly in metastatic settings. The landmark KEYNOTE-355 trial demonstrated that Pembrolizumab combined with chemotherapy significantly improved progression-free survival (PFS) and overall survival (OS) in patients with PD-L1-positive metastatic TNBC (combined positive score [CPS] ≥10), with median OS reaching 23.0 months versus 16.1 months for chemotherapy alone (13). This benefit was attributed to Pembrolizumab’s ability to enhance antitumor immunity by reversing T-cell exhaustion, particularly in tumors with preexisting immune infiltration. In the neoadjuvant KEYNOTE-522 trial, Pembrolizumab added to chemotherapy doubled pathologic complete response (pCR) rates (64.8% vs. 51.2%) and reduced recurrence risk by 37%, establishing it as a standard for high-risk early-stage TNBC regardless of PD-L1 status (14, 15). While immune-related adverse events (irAEs) like thyroid dysfunction and pneumonitis occur in ∼15–20% of patients, these are generally manageable, supporting Pembrolizumab’s favorable risk-benefit profile (16).

Atezolizumab, a PD-L1 inhibitor, initially showed promise in metastatic TNBC through the IMpassion-130 trial, where its combination with nab-paclitaxel improved median OS in PD-L1-positive patients (25.4 vs. 17.9 months) (17). This benefit was linked to Atezolizumab’s modulation of the tumor microenvironment, including increased CD8^+^ T-cell activation and dendritic cell maturation (18). However, subsequent trials revealed context-dependent efficacy. In IMpassion131, Atezolizumab failed to enhance outcomes when paired with paclitaxel, suggesting that chemotherapy backbone selection critically influences immunotherapy efficacy (19). Similarly, in early-stage TNBC, the neoadjuvant IMpassion-031 trial reported a pCR rate of 58% with Atezolizumab versus 41% with chemotherapy alone, but long-term survival data remain pending (15). Despite these challenges, Atezolizumab remains a viable option for PD-L1-enriched TNBC, particularly in taxane-resistant settings, though its utility is overshadowed by Pembrolizumab’s broader approval and more robust clinical evidence.

Both Pembrolizumab and Atezolizumab have redefined TNBC treatment by integrating immunotherapy into standard chemotherapy regimens. Pembrolizumab’s success in both metastatic and neoadjuvant settings underscores its versatility, while Atezolizumab’s niche utility emphasizes the importance of biomarker-driven patient selection (14, 17). However, highlights challenges persist, including optimal sequencing of therapies, managing resistance in PD-L1-negative tumors, and minimizing irAEs. By addressing these challenges, next-generation immunotherapies may achieve durable remissions and redefine standards of care for TNBC patients.

### 2.2 CTLA-4 inhibitors

Cytotoxic T-lymphocyte-associated antigen 4 (CTLA-4) is a critical immune checkpoint receptor that suppresses T-cell activation by competitively binding to CD80/CD86 on antigen-presenting cells, thereby outcompeting the co-stimulatory receptor CD28. This mechanism inhibits early T-cell responses and promotes regulatory T-cell (Treg) activity, leading to the formation of an immunosuppressive tumor microenvironment (TME) (20). Therapeutic strategies targeting CTLA-4 aim to block its inhibitory signaling, thereby reactivating anti-tumor immunity. For example, the monoclonal antibody Ipilimumab (anti-CTLA-4) disrupts CTLA-4-CD80/CD86 interactions, promoting T-cell activation and reducing Treg-mediated suppression (21). In TNBC, which is characterized by deficient hormone receptor expression and poor prognosis, CTLA-4 blockade has shown promise in reshaping the immunosuppressive TME. Navarrete-Bernal et al. (21) demonstrated that CTLA-4-expressing TNBC cells exhibit activate the ERK1/2 and AKT pathways, which drive proliferation; Ipilimumab suppressed these pathways *in vitro*, resulting in tumor growth. Similarly, Rahman et al. (22) found that alkalization of the acidic TME with sodium bicarbonate enhanced the effects of anti-PD-L1/CTLA-4 therapy, increasing T-cell infiltration and cytokine production.

However, heterogeneity in CTLA-4 expression and immune evasion mechanisms remain significant challenges. For instance, Hussein et al. (23) identified NEAT1 long non-coding RNA (lncRNA) as a regulator of CTLA-4 and CD80, indicating that epigenetic modulation may contribute to resistance mechanisms. Clinical trials, such as Li et al.’s phase II study (24) of KN046 combined with nab-paclitaxel, revealed an objective response rate of 44% in metastatic TNBC, with PD-L1-positive patients exhibiting prolonged survival. These findings highlight the importance of CTLA-4 as a therapeutic target, yet emphasize the need for biomarker-driven approaches to overcome resistance.

### 2.3 LAG-3 inhibitors

LAG-3 (lymphocyte-activation gene 3) is an inhibitory immune checkpoint receptor expressed on activated T cells, natural killer (NK) cells, and regulatory T cells (Tregs). Its primary mechanism involves binding to MHC class II molecules and fibrinogen-like protein 1 (FGL1), resulting in the suppression of T-cell activation and cytokine secretion, as well as the promotion of Treg-mediated immunosuppression (25, 26). Unlike PD-1 and CTLA-4, LAG-3 employs unconventional signaling motifs, which complicates its functional characterization. Preclinical studies highlight its synergy with PD-1 in driving T-cell exhaustion, a phenomenon validated by the FDA-approved combination of Relatlimab (anti-LAG-3) and Nivolumab (anti-PD-1) for advanced melanoma, which demonstrated a 25% reduction in the risk of progression-free survival (27). However, LAG-3’s dual role—as both an immunosuppressor and a potential prognostic biomarker—underscores the need for context-specific evaluation, particularly in tumors with high MHC II or FGL1 expression (28).

In TNBC, LAG-3 expression exhibits paradoxical prognostic and therapeutic implications. A meta-analysis of 5,859 breast cancer patients revealed that high LAG-3(+) tumor-infiltrating lymphocyte (TIL) density correlates with improved OS in TNBC but not HER2-positive subtypes, suggesting a subtype-specific immunomodulatory role (29). Notably, LAG-3 and PD-L1 co-expression occurs in approximately46.5% of TNBC cases, indicating potential synergistic resistance mechanisms (30). Preclinical models demonstrate that dual LAG-3/PD-1 blockade reduces tumor growth, yet clinical trials in TNBC remain sparse. Intriguingly, low LAG-3 expression in TNBC patients receiving neoadjuvant chemotherapy (NAC) correlates with higher pCR rates, positioning LAG-3 as a predictive biomarker for NAC efficacy (31).

LAG-3’s dual identity—as a biomarker of favorable prognosis in TNBC and a mediator of immunosuppression—complicates its therapeutic targeting. While Hu et al. (29) caution against anti-LAG-3 monotherapy in TNBC, combination strategies (e.g., LAG-3/PD-1 co-blockade) hold promise, as evidenced by enhanced cytotoxic T-cell responses in melanoma (28). Ultimately, LAG-3’s therapeutic potential lies in precision approaches tailored to molecular and immune context.

### 2.4 TIGIT inhibitors

TIGIT (T cell immunoreceptor with immunoglobulin and ITIM domain) is an inhibitory checkpoint receptor expressed on activated T cells, NK cells, and regulatory T cells. It binds to CD155 (PVR) and CD112 (PVRL2), ligands overexpressed on tumor cells and antigen-presenting cells, thereby suppressing anti-tumor immunity (32). Mechanistically, TIGIT competes with the costimulatory receptor CD226 for CD155 binding, resulting in the inhibition of CD8^+^ T cell activation and cytokine production. Additionally, TIGIT recruits phosphatases like SHIP-1 to suppress downstream signaling pathways such as NF-κB and ERK, leading to T cell exhaustion (33). These findings have spurred clinical development of anti-TIGIT antibodies, including Tiragolumab (phase III), Vibostolimab (phase II), and Domvanalimab (phase III), often combined with PD-1/PD-L1 inhibitors (Table 1).

**TABLE 1:** TIGIT inhibitors in clinical development.

Drugs	Study findings	Study participants	Phase	References (author, year)
Tiragolumab	Improved PFS in NSCLC when combined with Atezolizumab	135 patients	III	(34)
Vibostolimab	Synergistic effect with Pembrolizumab in advanced solid tumors, enhancing anti-tumor T cell responses	Ongoing trials	II	(35)
Domvanalimab	Enhanced CD8^+^ T cell activity and tumor control in combination with Zimberelimab	300+ patients	III	(36)

NSCLC, non-small cell lung cancer.

In TNBC, a subtype with limited therapeutic options, TIGIT/CD155 axis dysregulation correlates with immune evasion. High TIGIT + immune cell infiltration and CD155 tumor expression are associated with poor prognosis and suppressed CD8^+^ T cell activity (37, 38). Preclinically, TIGIT blockade in TNBC models reverses T cell exhaustion and restores glucose metabolism via PI3K/AKT/mTOR pathway modulation, enhancing cytokine production and tumor control (38). These findings underscore TIGIT’s broader applicability in immunologically ‘cold’ tumors, where dual checkpoint targeting may overcome resistance mechanisms (39).

**FIGURE 1:**
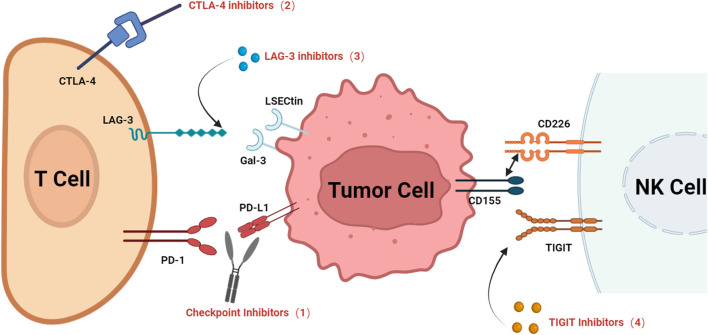
Emerging immune checkpoint targets and appropriate drugs in TNBC. The schematic shows several major abnormal signaling pathways, inhibited receptors and other key molecules involved in proliferation and progression in TNBC. The numbers represent the following immune checkpoint inhibitors:(1) PD-1/PD-L1 inhibitors (Pembrolizumab, Atezolizumab); (2) CTLA-4 inhibitors (Ipilimumab); (3) LAG-3 inhibitors (Relatlimab); (4) TIGIT inhibitors (Tiragolumab, Vibostolimab, Domvanalimab).

ICIs have reshaped TNBC treatment but face significant limitations rooted in tumor complexity. PD-1/PD-L1 inhibitors show efficacy in PD-L1-positive patients when paired with chemotherapy, yet their utility is hampered by biomarker variability and resistance mechanisms in PD-L1-negative tumors (14, 17). CTLA-4-targeted therapies encounter TNBC-specific barriers, including metabolic adaptations like glycolysis-driven immunosuppression and epigenetic regulation via non-coding RNAs, prompting the exploration of dual-target agents (e.g., CTLA-4/PD-L1 bispecifics) or metabolic inhibitors (23, 40). Emerging targets like LAG-3 and TIGIT present dual challenges: LAG-3’s paradoxical roles as both a prognostic marker and immunosuppressive mediator necessitate refined targeting strategies (e.g., FGL1 inhibition) and standardized diagnostic criteria (41, 42), while TIGIT blockade risks unintended Treg activation, demanding careful integration with therapies such as PARP inhibitors to optimize efficacy (43, 44). Collectively, these hurdles underscore the need for biomarker innovation, combination therapies tailored to TNBC’s unique biology, and strategies addressing dynamic immune-microenvironment interactions (Figure 1).

## 3 Beyond checkpoint blockade: CAR-T cells and cancer vaccines

While ICIs have revolutionized TNBC treatment by reactivating exhausted T cells, they primarily rely on the existence of a pre-existing antitumor immune response. To address tumors with low immunogenicity or immune-excluded microenvironments, therapeutic strategies aimed at generating *de novo* tumor-specific immunity, such as chimeric antigen receptor (CAR)-T cells and cancer vaccines, are under intensive investigation (45). CAR-T cell therapy involves engineering a patient’s own T cells to recognize and eliminate tumor cells expressing a predefined surface antigen. In TNBC, promising preclinical targets include ROR1, B7-H3, and mesothelin (46, 47). However, the translation of CAR-T therapy to solid tumors like TNBC faces formidable obstacles. These include the identification of truly tumor-specific antigens to mitigate on-target/off-tumor toxicity, the highly immunosuppressive TME that suppresses CAR-T cell infiltration, persistence, and effector function, and the inherent heterogeneity of antigen expression within and between tumors (48, 49). For instance, recent research highlights that host factors like obesity can further impair CAR-T cell metabolic fitness and memory formation, thereby compromising antitumor efficacy (48). To overcome these barriers, next-generation strategies are exploring combination approaches, such as engineering CAR-T cells to resist TME-mediated suppression or combining them with oncolytic viruses or ICIs to enhance their activity (47, 49).

Cancer vaccines represent another pivotal approach to ignite antitumor immunity by priming or amplifying endogenous T-cell responses against tumor-associated antigens (TAAs) or tumor-specific neoantigens. Early vaccine strategies in TNBC targeted overexpressed TAAs like p53, MUC1, or survivin, but their clinical efficacy has been limited by central tolerance and the risk of antigen-negative escape (50, 51). To overcome tumor heterogeneity and immune evasion, the field has shifted toward multi-antigen and personalized neoantigen vaccines. A novel multi-antigen peptide vaccine (TNBCvax) targeting TOP2A, HIF-1α, and IGF-1R demonstrated significant tumor reduction and robust CD8^+^ T-cell infiltration in preclinical TNBC models, highlighting the potential of targeting multiple drivers simultaneously to prevent escape (52). Concurrently, the advent of mRNA technology and advanced bioinformatics has accelerated the development of personalized neoantigen vaccines. These platforms can rapidly encode patient-specific mutations, and a first-in-human trial of a neoantigen DNA vaccine in TNBC has already validated its safety and immunogenicity (53, 54). Despite this progress, challenges persist in accurately predicting which neoantigens will elicit a potent and durable immune response, and in overcoming the immunosuppressive TME that can render vaccines ineffective (55, 56).

In essence, ICIs function by “releasing the brake” on existing, often exhausted, tumor-specific T cells within the TME. In contrast, CAR-T cells and cancer vaccines aim to “step on the gas” by generating a new or amplified wave of tumor-specific immunity, either through adoptive transfer of potent effector cells or through *in vivo* education of the patient’s own immune system. These modalities are not mutually exclusive but are potentially synergistic. A future paradigm may involve using vaccines or CAR-T cells to establish or expand a pool of tumor-specific T cells, followed by ICIs to sustain their function by preemptively blocking checkpoint-mediated exhaustion within the hostile TME (55, 57). While their current role in TNBC management remains largely investigational, the rapid pace of innovation in target discovery, cell engineering, and delivery platforms holds immense promise for transforming these approaches into clinically viable options.

## 4 Combination strategies

TNBC remains a therapeutic challenge due to its aggressive biology, lack of targetable receptors, and heterogeneity. While chemotherapy has been the cornerstone of treatment, emerging combination strategies aim to amplify efficacy by leveraging synergistic mechanisms, overcoming resistance, and tailoring therapies to molecular vulnerabilities. Recent advances in immunotherapy, targeted agents, and ADCs have redefined the therapeutic landscape, offering hope for improved outcomes. This section explores key combination approaches, including immune checkpoint inhibitors with chemotherapy, PARP inhibitors, ADCs, and dual immune checkpoint blockade, highlighting their clinical impact and future potential.

### 4.1 ICIs with chemotherapy

The combination of ICIs and chemotherapy in TNBC leverages synergistic mechanisms to enhance antitumor immunity. Chemotherapy induces immunogenic cell death (ICD), releasing tumor-associated antigens (TAAs) and damage-associated molecular patterns (DAMPs), which promote dendritic cell maturation and T-cell priming (58). Meanwhile, ICIs block immune evasion by inhibiting the PD-1/PD-L1 axis, thereby reinvigorating exhausted T cells and amplifying cytotoxic T-cell activity (6). Preclinical studies suggest chemotherapy upregulates PD-L1 expression on tumor cells, creating a feedback loop that ICIs can disrupt (59). Additionally, chemotherapy reduces immunosuppressive elements in the tumor microenvironment, such as regulatory T cells and myeloid-derived suppressor cells, further enhancing ICI efficacy (60). This dual approach transforms “cold” TNBC tumors into “hot” immunogenic environments, priming systemic and durable responses (61).

Recent trials demonstrate significant improvements in outcomes with ICI-chemotherapy combinations, although the magnitude of benefit varies across study designs. In the neoadjuvant setting, the KEYNOTE-522 trial showed that adding pembrolizumab to carboplatin/paclitaxel followed by anthracyclines increased pathological complete response rates from 51% to 65% and improved 3-year event-free survival to 85% versus 77% with chemotherapy alone (62, 63). In metastatic disease, KEYNOTE-355 established pembrolizumab plus chemotherapy as a standard for PD-L1-positive (CPS ≥10) TNBC, demonstrating a significant overall survival benefit (64). In contrast, IMpassion130 reported only a modest progression-free survival gain with atezolizumab plus nab-paclitaxel, and its companion trial IMpassion131 failed to confirm benefit when atezolizumab was combined with paclitaxel instead of nab-paclitaxel (65). These divergent outcomes underscore the critical influence of chemotherapy backbone selection—particularly the choice between nab-paclitaxel and paclitaxel—and PD-L1 assessment methodology on ICI efficacy (66). Beyond these agents, emerging data support the activity of novel ICIs such as toripalimab and adebrelimab in combination with chemotherapy, with manageable safety profiles (59, 67). Notably, although ICI-chemotherapy combinations increase the incidence of grade ≥3 adverse events, treatment-related mortality remains low, and immune-related adverse events are generally manageable with standard protocols (66). Collectively, the integration of ICIs with chemotherapy represents a paradigm shift in TNBC management, particularly for high-risk early-stage and metastatic disease. By converting immunologically inert tumors into responsive ones, this strategy directly addresses TNBC’s aggressive biology and lack of targeted therapies (68). These advances underscore the transformative potential of immunotherapy in TNBC and warrant continued innovation.

### 4.2 PARP inhibitors and immunotherapy

The interplay between PARP inhibitors (PARPis) and immunotherapy in TNBC hinges on the modulation of DNA damage response (DDR) pathways and immune activation. PARPis induce synthetic lethality in BRCA-mutated tumors by blocking single-strand DNA repair, leading to replication stress and genomic instability. This accumulation of DNA damage triggers the cGAS-STING pathway, promoting type I interferon secretion and dendritic cell activation, which enhances T-cell infiltration and antitumor immunity (69, 70). Additionally, PARP inhibition upregulates PD-L1 expression on tumor cells and reduces immunosuppressive myeloid-derived suppressor cells (MDSCs), creating a favorable microenvironment for ICIs (71, 72). Preclinical studies further suggest that PARPis increase tumor mutational burden and neoantigen presentation, priming tumors for immune recognition (73). These mechanisms collectively establish a rationale for combining PARPis with ICIs to overcome TNBC’s immunosuppressive milieu.

Clinical trials have demonstrated promising outcomes for PARPi-ICI combinations in TNBC. In BRCA-mutated metastatic TNBC, the phase II MEDIOLA trial reported that olaparib plus durvalumab achieved an objective response rate of 63% (72). Similarly, the TOPACIO/KEYNOTE-162 trial evaluated niraparib plus pembrolizumab in metastatic TNBC, showing durable responses in both BRCA-mutant and BRCA-wild-type cohorts, with a disease control rate of 49% and manageable toxicity (74). Ongoing studies continue to explore the synergistic potential of this combination across different TNBC subgroups (75). The integration of PARPis and immunotherapy represents a paradigm shift in TNBC management, offering durable responses in traditionally chemoresistant subsets.

### 4.3 Antibody-drug conjugates (ADCs) and immunotherapy

The integration of antibody-drug conjugates (ADCs) and immunotherapy in TNBC leverages complementary mechanisms to enhance antitumor efficacy. ADCs, such as TROP2-targeted agents (e.g., Sacituzumab Govitecan), deliver cytotoxic payloads directly to tumor cells, inducing immunogenic cell death (ICD) and releasing tumor-associated antigens (TAAs) that prime dendritic cells and activate CD8^+^ T cells (76, 77). Preclinical models further demonstrate that ADCs targeting CD276/B7-H3 enhance T-cell cytotoxicity by blocking coinhibitory signals, suggesting a dual role for ADCs in payload delivery and immune modulation (78). These mechanisms underscore the potential of combining ADCs with ICIs to amplify both direct tumors killing and adaptive immune responses.

Emerging clinical trials highlight the promise of ADC-immunotherapy combinations in TNBC. The phase III TROPION-Breast02 trial demonstrated that datopotamab deruxtecan (Dato-DXd) monotherapy achieved a 31.8% objective response rate (ORR) in metastatic TNBC, with responders showing enriched tumor-infiltrating lymphocytes (TILs), suggesting inherent immunogenicity that could be further exploited with ICIs (77, 79). Similarly, the NeoSTAR trial evaluated neoadjuvant Sacituzumab Govitecan, achieving a 30% pCR rate, with higher Ki-67 and TILs correlating with improved outcomes, indicating a potential role for adjuvant immunotherapy to eliminate residual disease (80). These advances underscore the feasibility of combining ADCs with ICIs to extend survival in both early and metastatic TNBC.

### 4.4 Dual immune checkpoint inhibition

Dual immune checkpoint inhibition, targeting both PD-1/PD-L1 and other pathways such as CTLA-4, has shown potential in TNBC. This approach aims to overcome immune resistance by simultaneously targeting multiple inhibitory pathways.

The KEYNOTE-522 trial established Pembrolizumab combined with chemotherapy as a standard neoadjuvant regimen, achieving a 64.8% pCR rate in early TNBC (81). Building on this, dual ICI strategies are being explored to overcome resistance. For example, the phase II MORPHEUS-TNBC trial evaluated Atezolizumab with Tiragolumab, demonstrating a 37% ORR in metastatic TNBC, with responders exhibiting elevated CD8^+^ T-cell infiltration and reduced PD-L1+ myeloid cells (36). Similarly, early-phase trials of bintrafusp alfa (TGF-β/PD-L1 bifunctional inhibitor) in TNBC showed prolonged disease control in PD-L1-low tumors, suggesting TGF-β blockade mitigates stromal barriers to T-cell penetration (82).

These results suggest that dual immune checkpoint inhibition may offer a new avenue for TNBC treatment, particularly in patients with high TMB and PD-L1 expression. However, toxicity remains a challenge; grade ≥3 irAEs occur in approximately30% of patients, necessitating careful patient selection and biomarker-driven approaches (66, 81) (Table 2).

**TABLE 2:** Other combination therapies for TNBC.

Drugs	Study findings	Study participants	Phase	References (author, year)
Pembrolizumab + Chemo	Increased pCR (65%) and 3-year EFS (85%) in early-stage TNBC	1,174 patients	III	(64)
Durvalumab + Chemo	Enhanced 3-year EFS (85.6% vs. 77.2%) despite modest pCR improvement	174 patients	II	(83)
Eganelisib + Atezolizumab + Chemo	Promoted macrophage reprogramming and immune activation in metastatic TNBC	23 patients	II	(84)
Niraparib + Dostarlimab	Improved PFS in HRD-positive TNBC; enhanced CD8^+^ T-cell infiltration	Metastatic TNBC (n = 45)	II	(85)
Veliparib + Atezolizumab	Synergistic STING activation; 40% ORR in PD-L1+ TNBC	Advanced TNBC (n = 32)	I/II	(69)
Rucaparib + Camrelizumab	Enhanced DNA damage and PD-L1 upregulation; 35% clinical benefit rate	Refractory TNBC (n = 50)	II	(86)
Spartalizumab + LAG525	Increased T-cell activation; manageable toxicity in refractory TNBC	Phase I solid tumor patients	I	(87)
INCAGN02385 + Nivolumab	Synergistic reduction of exhausted T cells; preliminary antitumor activity	Early-stage TNBC models	Preclinical	(81)

Abbreviations: Chemo, chemotherapy; LAG525, anti-LAG-3; INCAGN02385, anti-TIM-3.

**FIGURE 2:**
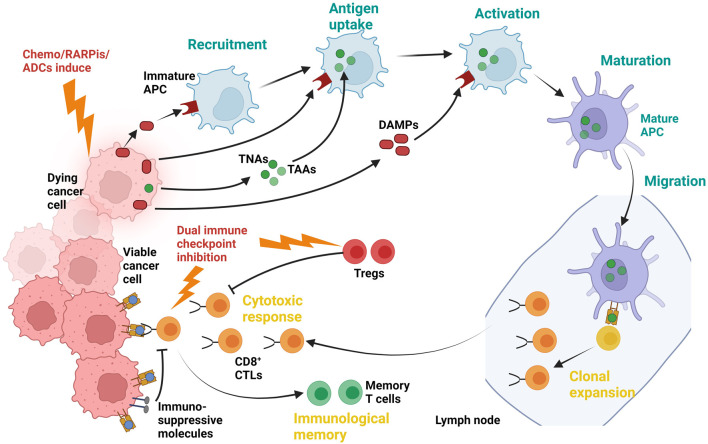
The combined action of chemotherapy, PARPis, and ADCs triggers immunogenic death of cancer cells, releasing TAAs and DAMPs. These signals activate immature APCs, which undergo maturation and migrate to lymph nodes to prime CD8^+^ cytotoxic T lymphocytes (CTLs). Dual immune checkpoint inhibition counteracts immunosuppressive molecules (e.g., PD-1/PD-L1, CTLA-4) and suppresses Tregs, unleashing CTLs to mount a robust cytotoxic response against viable cancer cells.

Combination therapies in TNBC aim to overcome drug resistance and boost immune attacks on tumors. By pairing treatments like dual immune checkpoint inhibitors with drugs that break down the tumor’s defenses, these strategies turn “cold” tumors into “hot” ones, attracting more immune cells to fight cancer. While early trials show improved response rates, challenges like severe side effects and unpredictable patient outcomes remain. Not all tumors respond the same way, and current tools cannot reliably predict who will benefit. This gap highlights the urgent need to identify biomarkers—specific molecular or immune signals—that guide treatment choices. Understanding these biomarkers will help match the right combinations to the right patients, minimizing risks and maximizing long-term survival (Figure 2).

## 5 Potential biomarkers for precision medicine

The development of biomarkers is critical for optimizing treatment strategies and identifying patients who may benefit most from specific therapies. Recent advancements have increasingly focused on identifying biomarkers that predict response to immunotherapy, targeted therapies, and combination regimens.

### 5.1 PD-L1 expression

PD-L1 is a transmembrane glycoprotein that binds to PD-1 receptors on T cells, delivering inhibitory signals to suppress cytotoxic T-cell activity, thereby enabling tumor immune evasion. In TNBC, PD-L1 is frequently overexpressed on tumor cells and tumor-associated immune cells, creating an immunosuppressive microenvironment that promotes cancer progression (88, 89).

PD-L1 expression has emerged as a predictive biomarker for ICI efficacy. The IMpassion130 and KEYNOTE-355 trials demonstrated that combining anti-PD-L1/PD-1 agents (Atezolizumab, Pembrolizumab) with chemotherapy significantly PFS and OS in PD-L1-positive metastatic TNBC (90, 91). However, PD-L1 testing remains controversial due to spatial and temporal heterogeneity, with only 40%–60% of TNBCs expressing PD-L1. Emerging strategies integrate PD-L1 with tumor mutational burden (TMB) or interferon-gamma signatures to refine patient selection (92). While current PD-L1-based therapies have transformed TNBC management, challenges such as heterogeneity and resistance persist.

### 5.2 Tumor-infiltrating lymphocytes (TILs)

Tumor-infiltrating lymphocytes (TILs) are a key component of the tumor microenvironment and have emerged as a predictive biomarker for immunotherapy response in TNBC. TILs, particularly CD8^+^ cytotoxic T cells, reflect pre-existing anti-tumor immunity. High TIL levels correlate with enhanced chemotherapy response and prolonged survival in TNBC. TILs promote tumor cell lysis via cytokine release and direct cytotoxicity, while also enhancing antigen presentation (93, 94). High levels of TILs suggest an active immune response against the tumor, which correlates with better outcomes in patients treated with ICIs (92).

The presence of TILs serves not only as a prognostic marker but also as a dynamic indicator of treatment response. Studies have shown that changes in TIL density during treatment may predict immunotherapy efficacy. Retrospective analyses of neoadjuvant trials (e.g., GeparSixto) show that high stromal TILs (>30%) predict pCR rates exceeding 60% in TNBC. TIL quantification is now recommended in clinical guidelines for prognostication (92). However, heterogeneous TILs distribution within tumors and the absence of standardized quantification methods remain challenges in clinical implementation (89).

### 5.3 Tumor mutational burden (TMB)

Tumor mutational burden (TMB) is a promising biomarker for predicting immunotherapy response in TNBC. TMB measures the total number of mutations in a tumor genome, and high TMB is associated with the presence of neoantigens that can be recognized by the immune system, potentiating antitumor immunity (89). In TNBC, high TMB has been linked to enhanced responses to ICIs, as these tumors are more likely to elicit a robust immune response due to the presence of numerous neoantigens (95).

High TMB has been identified as a predictive biomarker for ICI efficacy. In the KEYNOTE-119 trial, TNBC patients with high TMB showed improved response rates to Pembrolizumab compared to those with low TMB (92). Additionally, TMB has been integrated into multi-omics models to stratify TNBC patients for immunotherapy. For example, the FUSCC classification system uses TMB alongside immune gene signatures to identify immunomodulatory TNBC subtypes that benefit most from ICIs (96). Despite the promise of TMB, its clinical utility is limited by differences in measurement methods and thresholds, so future research should focus on standardization of TMB assessment (95).

### 5.4 Circulating tumor DNA (ctDNA)

Circulating tumor DNA (ctDNA) consists of small DNA fragments shed by tumor cells into the bloodstream. In TNBC, ctDNA carries tumor-specific mutations, copy number alterations, and epigenetic changes, providing a non-invasive method to monitor tumor dynamics and heterogeneity (97, 98).

ctDNA has shown promise in detecting minimal residual disease (MRD) after surgery or chemotherapy. Studies have demonstrated that ctDNA positivity post-treatment correlates with a higher risk of recurrence, enabling early intervention (97). Additionally, ctDNA analysis can identify actionable mutations (e.g., PIK3CA, TP53) and guide targeted therapies. For example, the FUTURE-C-Plus trial used ctDNA to monitor treatment response in TNBC patients receiving combination immunotherapy (96). Future research should focus on developing standardised ctDNA assays and exploring their role in real-time therapeutic adaptation, integrating them with other biomarkers (e.g., TILs, PD-L1) to improve predictive accuracy.

### 5.5 Exosomes and MicroRNAs (miRNAs)

Exosomes are extracellular vesicles that carry proteins, lipids, and nucleic acids, including miRNAs. In TNBC, exosomal miRNAs (e.g., miR-21, miR-155) regulate tumor progression, metastasis, and immune evasion by modulating gene expression in recipient cells (98, 99).

Exosomal miRNAs have emerged as promising diagnostic and prognostic biomarkers. For example, miR-21 is overexpressed in TNBC and correlates with poor survival, while miR-155 is associated with immune suppression (99). Additionally, exosomes can deliver therapeutic agents or immune modulators (98), offering a novel approach to TNBC treatment.

The exploration of biomarkers in TNBC has advanced precision medicine, yet the heterogeneous nature of the disease limits the predictive power of any single marker. Although PD-L1, TILs, TMB, and ctDNA each hold clinical value, their performance varies across populations, reflecting the complex interplay between tumor biology and immune dynamics. An emerging consensus supports integrating multiple biomarkers into combinatorial models to improve predictive accuracy—for example, combining PD-L1 with TIL density or immune gene signatures has shown superior stratification of immunotherapy responders. Advances in multi-omics and machine learning now enable the development of such composite panels. Their clinical implementation, supported by standardized assays, promises to transition TNBC management toward truly personalized immunotherapy.

## 6 Challenges and future directions

### 6.1 Overcoming tumor heterogeneity and resistance

TNBC is highly diverse, with different tumors showing unique molecular features. This diversity makes it difficult to find treatments that work for everyone. For example, some tumors develop resistance to drugs like PARPis by reactivating DNA repair mechanisms, while others evade immune system attacks by suppressing immune signals (100).

A major clinical challenge in TNBC is the development of chemoresistance, a multifaceted process driven by tumor-intrinsic factors such as the enrichment of cancer stem cells (CSCs) and the activity of ATP-binding cassette (ABC) transporters that actively efflux cytotoxic drugs (101). These mechanisms not only limit the efficacy of conventional chemotherapy but also shape a more aggressive and immunosuppressive tumor microenvironment. To counter this, biomarker-driven strategies have emerged as a critical approach to enhance drug efficacy in chemo resistant disease. PARP inhibitors like Olaparib exploit synthetic lethality in tumors with germline *BRCA1/2* mutations, offering a targeted option that remains effective even after chemotherapy failure, while antibody-drug conjugates such as Sacituzumab govitecan circumvent classical resistance mechanisms by delivering cytotoxic payloads directly to Trop-2-expressing tumor cells (101). Importantly, these agents are not merely alternatives to chemotherapy; they also exhibit immunomodulatory properties—PARP inhibitors can activate the cGAS-STING pathway to enhance tumor immunogenicity, and ADC-induced immunogenic cell death may prime antitumor immune responses. Moving forward, the challenge is not simply to overcome chemoresistance in isolation, but to integrate these targeted agents into rational combination regimens that simultaneously re-sensitize tumors to immunotherapy. This will require prospective identification of predictive biomarkers—including BRCA mutation status, homologous recombination deficiency scores, and target antigen expression—to guide not only patient selection but also the optimal sequencing of therapies that can both bypass resistance and remodel the tumor microenvironment in favor of durable immune control.

### 6.2 Expanding access to advanced therapies

Many cutting-edge treatments, such as ADCs and immunotherapy, are expensive and require specialized infrastructure, making them inaccessible in low-resource settings. For example, Sacituzumab Govitecan is not widely available in developing countries due to cost and logistical challenges (102).

To bridge this gap, researchers are developing cost-effective alternatives, such as simplified biomarker tests and generic versions of ADCs. Global initiatives, like the WHO’s Global Breast Cancer Initiative, aim to improve access to these therapies by subsidizing costs and training healthcare providers. Telemedicine and decentralized clinical trials could also bring advanced treatments to underserved populations (103).

### 6.3 Developing next-generation therapies

Current treatments like ADCs and CAR-T cell therapy face limitations, such as toxicity and difficulty targeting solid tumors. For example, ADCs can damage healthy tissues, while CAR-T cells struggle to penetrate and persist in TNBC tumors (104). Next-generation therapies are being designed to overcome these challenges. New ADCs with more stable linkers and dual-targeting capabilities could reduce side effects and improve efficacy. CAR-T cells engineered with chemokine receptors or combined with oncolytic viruses may better infiltrate tumors and stimulate immune responses (78, 105). These innovations could make immunotherapy more effective for TNBC patients.

In conclusion, while TNBC remains a challenging disease, advancements in immune checkpoint inhibitors, combination therapies, and biomarker identification offer hope for improved outcomes. Ongoing research and collaboration are essential to overcome current limitations and develop more effective and personalized treatments for TNBC patients.

## References

[1] Won KA Spruck C . Triple-negative breast cancer therapy: current and future perspectives. Int J Oncol (2020) 57(6):1245–61. 10.3892/ijo.2020.5135 33174058 PMC7646583

[2] Abdou Y Goudarzi A Yu JX Upadhaya S Vincent B Carey LA . Immunotherapy in triple negative breast cancer: beyond checkpoint inhibitors. NPJ Breast Cancer (2022) 8(1):121. 10.1038/s41523-022-00486-y 36351947 PMC9646259

[3] Zagami P Carey LA . Triple negative breast cancer: pitfalls and progress. NPJ Breast Cancer (2022) 8(1):95. 10.1038/s41523-022-00468-0 35987766 PMC9392735

[4] Sukumar J Gast K Quiroga D Lustberg M Williams N . Triple-negative breast cancer: promising prognostic biomarkers currently in development. Expert Rev Anticancer Ther (2021) 21(2):135–48. 10.1080/14737140.2021.1840984 33198517 PMC8174647

[5] Yang F Xiao Y Ding JH Jin X Ma D Li DQ Ferroptosis heterogeneity in triple-negative breast cancer reveals an innovative immunotherapy combination strategy. Cell Metab (2023) 35(1):84–100.e8. 10.1016/j.cmet.2022.09.021 36257316

[6] Luo C Wang P He S Zhu J Shi Y Wang J . Progress and prospect of immunotherapy for triple-negative breast cancer. Front Oncol (2022) 12:919072. 10.3389/fonc.2022.919072 35795050 PMC9251310

[7] Cortes J Haiderali A Huang M Pan W Schmid P Akers KG Neoadjuvant immunotherapy and chemotherapy regimens for the treatment of high-risk, early-stage triple-negative breast cancer: a systematic review and network meta-analysis. BMC Cancer (2023) 23(1):792. 10.1186/s12885-023-11293-4 37612624 PMC10463750

[8] Lotfinejad P Kazemi T Mokhtarzadeh A Shanehbandi D Jadidi Niaragh F Safaei S PD-1/PD-L1 axis importance and tumor microenvironment immune cells. Life Sci (2020) 259:118297. 10.1016/j.lfs.2020.118297 32822718

[9] Capuozzo M Celotto V Santorsola M Fabozzi A Landi L Ferrara F Emerging treatment approaches for triple-negative breast cancer. Med Oncol (2023) 41(1):5. 10.1007/s12032-023-02257-6 38038783

[10] Li Y Zhang H Merkher Y Chen L Liu N Leonov S Recent advances in therapeutic strategies for triple-negative breast cancer. J Hematol Oncol (2022) 15(1):121. 10.1186/s13045-022-01341-0 36038913 PMC9422136

[11] Yu Y Jin X Zhu X Xu Y Si W Zhao J . PD-1/PD-L1 immune checkpoint inhibitors in metastatic triple-negative breast cancer: a systematic review and meta-analysis. Front Immunol (2023) 14:1206689. 10.3389/fimmu.2023.1206689 37377959 PMC10292799

[12] Lin X Kang K Chen P Zeng Z Li G Xiong W Regulatory mechanisms of PD-1/PD-L1 in cancers. Mol Cancer (2024) 23(1):108. 10.1186/s12943-024-02023-w 38762484 PMC11102195

[13] Cortes J Cescon DW Rugo HS Nowecki Z Im SA Yusof MM Pembrolizumab plus chemotherapy versus placebo plus chemotherapy for previously untreated locally recurrent inoperable or metastatic triple-negative breast cancer (KEYNOTE-355): a randomised, placebo-controlled, double-blind, phase 3 clinical trial. Lancet (2020) 396(10265):1817–28. 10.1016/S0140-6736(20)32531-9 33278935

[14] Cortes J Rugo HS Cescon DW Im SA Yusof MM Gallardo C Pembrolizumab plus chemotherapy in advanced triple-negative breast cancer. N Engl J Med (2022) 387(3):217–26. 10.1056/NEJMoa2202809 35857659

[15] Rizzo A Cusmai A Acquafredda S Giovannelli F Rinaldi L Misino A KEYNOTE-522, IMpassion031 and GeparNUEVO: changing the paradigm of neoadjuvant immune checkpoint inhibitors in early triple-negative breast cancer. Future Oncol (2022) 18(18):2301–9. 10.2217/fon-2021-1647 35378995

[16] Khan M Du K Ai M Wang B Lin J Ren A PD-L1 expression as biomarker of efficacy of PD-1/PD-L1 checkpoint inhibitors in metastatic triple negative breast cancer: a systematic review and meta-analysis. Front Immunol (2023) 14:1060308. 10.3389/fimmu.2023.1060308 36949944 PMC10027008

[17] Emens LA Molinero L Loi S Rugo HS Schneeweiss A Diéras V Atezolizumab and nab-Paclitaxel in advanced triple-negative breast cancer: biomarker evaluation of the IMpassion130 study. J Natl Cancer Inst (2021) 113(8):1005–16. 10.1093/jnci/djab004 33523233 PMC8328980

[18] Bassez A Vos H Van DYCK Floris G Arijs I Desmedt C A single-cell map of intratumoral changes during anti-PD1 treatment of patients with breast cancer. Nat Med (2021) 27(5):820–32. 10.1038/s41591-021-01323-8 33958794

[19] Miles D Gligorov J André F Cameron D Schneeweiss A Barrios C Primary results from IMpassion131, a double-blind, placebo-controlled, randomised phase III trial of first-line paclitaxel with or without atezolizumab for unresectable locally advanced/metastatic triple-negative breast cancer. Ann Oncol (2021) 32(8):994–1004. 10.1016/j.annonc.2021.05.801 34219000

[20] Virassamy B Caramia F Savas P Sant S Wang J Christo SN Intratumoral CD8(+) T cells with a tissue-resident memory phenotype mediate local immunity and immune checkpoint responses in breast cancer. Cancer Cell (2023) 41(3):585–601.e8. 10.1016/j.ccell.2023.01.004 36827978

[21] Navarrete-Bernal MGC Cervantes-Badillo MG Martínez-Herrera JF Lara-Torres CO Gerson-Cwilich R Zentella-Dehesa A Biological landscape of triple negative breast cancers expressing CTLA-4. Front Oncol (2020) 10:1206. 10.3389/fonc.2020.01206 32850353 PMC7419680

[22] Rahman A Janic B Rahman T Singh H Ali H Rattan R Immunotherapy enhancement by targeting extracellular tumor pH in triple-negative breast cancer mouse model. Cancers (Basel) (2023) 15(20):4931. 10.3390/cancers15204931 37894298 PMC10605606

[23] Hussein NH Eissa RA De Bruyn M El Tayebi HM . NEAT1: culprit lncRNA linking PIG-C, MSLN, and CD80 in triple-negative breast cancer. Life Sci (2022) 299:120523. 10.1016/j.lfs.2022.120523 35378140

[24] Li Q Liu J Zhang Q Ouyang Q Zhang Y Liu Q The anti-PD-L1/CTLA-4 bispecific antibody KN046 in combination with nab-paclitaxel in first-line treatment of metastatic triple-negative breast cancer: a multicenter phase II trial. Nat Commun (2024) 15(1):1015. 10.1038/s41467-024-45160-y 38310192 PMC10838317

[25] Chocarro L Blanco E Zuazo M Arasanz H Bocanegra A Fernández-Rubio L Understanding LAG-3 signaling. Int J Mol Sci (2021) 22(10):5282. 10.3390/ijms22105282 34067904 PMC8156499

[26] Maruhashi T Sugiura D Okazaki IM Shimizu K Maeda TK Ikubo J Binding of LAG-3 to stable peptide-MHC class II limits T cell function and suppresses autoimmunity and anti-cancer immunity. Immunity (2022) 55(5):912–24.e8. 10.1016/j.immuni.2022.03.013 35413245

[27] Aggarwal V Workman CJ Vignali DAA . LAG-3 as the third checkpoint inhibitor. Nat Immunol (2023) 24(9):1415–22. 10.1038/s41590-023-01569-z 37488429 PMC11144386

[28] Cillo AR Cardello C Shan F Karapetyan L Kunning S Sander C Blockade of LAG-3 and PD-1 leads to co-expression of cytotoxic and exhaustion gene modules in CD8(+) T cells to promote antitumor immunity. Cell (2024) 187(16):4373–88.e15. 10.1016/j.cell.2024.06.036 39121849 PMC11346583

[29] Hu G Wang S Wang S Ding Q Huang L . LAG-3(+) tumor-infiltrating lymphocytes ameliorates overall survival in triple-negative breast cancer patients. Front Oncol (2022) 12:986903. 10.3389/fonc.2022.986903 36761428 PMC9904386

[30] Wu S Shi X Wang J Wang X Liu Y Luo Y Triple-negative breast cancer: intact mismatch repair and partial Co-Expression of PD-L1 and LAG-3. Front Immunol (2021) 12:561793. 10.3389/fimmu.2021.561793 33717059 PMC7943629

[31] Asano Y Kashiwagi S Takada K Ishihara S Goto W Morisaki T Clinical significance of expression of immunoadjuvant molecules (LAG-3, TIM-3, OX-40) in neoadjuvant chemotherapy for breast cancer. Anticancer Res (2022) 42(1):125–36. 10.21873/anticanres.15466 34969718

[32] Chauvin JM Zarour HM . TIGIT in cancer immunotherapy. J Immunother Cancer (2020) 8(2):e000957. 10.1136/jitc-2020-000957 32900861 PMC7477968

[33] Liu L Wang A Liu X Han S Sun Y Zhang J Blocking TIGIT/CD155 signalling reverses CD8(+) T cell exhaustion and enhances the antitumor activity in cervical cancer. J Transl Med (2022) 20(1):280. 10.1186/s12967-022-03480-x 35729552 PMC9210727

[34] Banta KL Xu X Chitre AS Au-Yeung A Takahashi C O'Gorman WE Mechanistic convergence of the TIGIT and PD-1 inhibitory pathways necessitates co-blockade to optimize anti-tumor CD8(+) T cell responses. Immunity (2022) 55(3):512–26.e9. 10.1016/j.immuni.2022.02.005 35263569 PMC9287124

[35] Howard FM Villamar D He G Pearson AT Nanda R . The emerging role of immune checkpoint inhibitors for the treatment of breast cancer. Expert Opin Investig Drugs (2022) 31(6):531–48. 10.1080/13543784.2022.1986002 34569400 PMC8995399

[36] Chu X Tian W Wang Z Zhang J Zhou R . Co-inhibition of TIGIT and PD-1/PD-L1 in cancer immunotherapy: mechanisms and clinical trials. Mol Cancer (2023) 22(1):93. 10.1186/s12943-023-01800-3 37291608 PMC10249258

[37] Boissière-Michot F Chateau MC Thézenas S Guiu S Bobrie A Jacot W . Correlation of the TIGIT-PVR immune checkpoint axis with clinicopathological features in triple-negative breast cancer. Front Immunol (2022) 13:1058424. 10.3389/fimmu.2022.1058424 36544779 PMC9760730

[38] Huang M Yu X Wang Q Jiang Z Li X Chen W The immune checkpoint TIGIT/CD155 promotes the exhaustion of CD8 + T cells in TNBC through glucose metabolic reprogramming mediated by PI3K/AKT/mTOR signaling. Cell Commun Signal (2024) 22(1):35. 10.1186/s12964-023-01455-z 38216949 PMC10785424

[39] Kraehenbuehl L Weng CH Eghbali S Wolchok JD Merghoub T . Enhancing immunotherapy in cancer by targeting emerging immunomodulatory pathways. Nat Rev Clin Oncol (2022) 19(1):37–50. 10.1038/s41571-021-00552-7 34580473

[40] Schreier A Zappasodi R Serganova I Brown KA Demaria S Andreopoulou E . Facts and perspectives: implications of tumor glycolysis on immunotherapy response in triple negative breast cancer. Front Oncol (2022) 12:1061789. 10.3389/fonc.2022.1061789 36703796 PMC9872136

[41] Heimes AS Almstedt K Krajnak S Runkel A Droste A Schwab R Prognostic impact of LAG-3 mRNA expression in early breast cancer. Biomedicines (2022) 10(10):2656. 10.3390/biomedicines10102656 36289918 PMC9599264

[42] Qian Y Sun Y Shi P Zhou X Zhang Q Dong Q Development of LAG-3/FGL1 blocking peptide and combination with radiotherapy for cancer immunotherapy. Acta Pharm Sin B (2024) 14(3):1150–65. 10.1016/j.apsb.2023.12.011 38486998 PMC10935467

[43] Zhou R Chen S Wu Q Liu L Wang Y Mo Y CD155 and its receptors in cancer immune escape and immunotherapy. Cancer Lett (2023) 573:216381. 10.1016/j.canlet.2023.216381 37660884

[44] Cai L Li Y Tan J Xu L . Targeting LAG-3, TIM-3, and TIGIT for cancer immunotherapy. J Hematol Oncol (2023) 16(1):101. 10.1186/s13045-023-01499-1 37670328 PMC10478462

[45] Choi Y Tan J Lin D Lee JS Yuan Y . Immunotherapy in breast cancer: beyond immune checkpoint inhibitors. Int J Mol Sci (2025) 26(8):3920. 10.3390/ijms26083920 40332761 PMC12027891

[46] Wong JKM Lam PY Coleborn E Jose J Alim L Tu C Development of a high-affinity anti-ROR1 variable region for broad anti-cancer immunotherapy. Mol Ther (2026) 34(3):1382–98. 10.1016/j.ymthe.2025.11.021 41338184 PMC12974153

[47] Alharbi S Qasem FF Talebi MT Omran NE Hamoudi R Harati R . Immunotherapy approaches for the treatment of triple-negative breast cancer. Cancers (Basel) (2026) 18(3):464. 10.3390/cancers18030464 41681936 PMC12897434

[48] Malian HM Pellegry CM Oh HM Glenny EM Ho AN Dotti G Obesity impairs the Antitumor activity of CAR-T cells in triple-negative breast cancer. bioRxiv (2025). 10.1101/2025.10.10.681694 41279207 PMC12632824

[49] Yan S Sun X Wang K . From cold to hot tumors: feasibility of applying therapeutic insights to TNBC. Discov Oncol (2025) 16(1):1942. 10.1007/s12672-025-03745-z 41117874 PMC12540225

[50] Rashid A Krishnan A Gupta S Gupta JC Talwar GP . Survivin targeting triple-fusion vaccine (DC)Survivin-LTB inhibits tumor growth in mouse model of triple-negative breast cancer. Med Oncol (2025) 43(1):35. 10.1007/s12032-025-03152-y 41343102

[51] Madhu Krishna B Garg P Ramisetty S Nair M Singhal SS . Recent advancements in immunotherapy for the treatment of metastatic breast cancer. Cancer Treat Res (2025) 129:33–65. 10.1007/978-3-031-97242-3_3 40847228

[52] Lee SB Qian J Pan J Sei S Wang Y You M . Immunoprevention of triple-negative breast cancer with a novel multivalent vaccine. Front Immunol (2025) 16:1638526. 10.3389/fimmu.2025.1638526 40969744 PMC12440889

[53] Zeng S Wang L Zeng W Sun L . Neoantigens in cancer immunoediting: from mechanisms to personalized vaccines in breast cancer. Front Cell Dev Biol (2025) 13:1733549. 10.3389/fcell.2025.1733549 41567982 PMC12816192

[54] Shatalov PA Bukaeva AA Veselovsky EM Traspov AA Bagdasarova DV Leukhina IA Neoantigen-driven immunotherapy in triple-negative breast cancer: emerging strategies and clinical potential. Biomedicines (2025) 13(9):2213. 10.3390/biomedicines13092213 41007774 PMC12467711

[55] Chatterjee A Chakraborty A Chatterjee S Pal S . Immunotherapy in triple-negative breast cancer: from molecular mechanisms to precision medicine-overcoming resistance and optimizing clinical outcomes. Crit Rev Oncol Hematol (2026) 221:105174. 10.1016/j.critrevonc.2026.105174 41724338

[56] Verma S Singh V Lang JE Gupta S . Reprogramming the immune microenvironment in triple-negative breast cancer with mRNA therapeutics. Cancer Lett (2026) 645:218350. 10.1016/j.canlet.2026.218350 41730382

[57] Székely B Pusztai L . Immunotherapy in the treatment of breast cancer. Magy Onkol (2025) 69(4):427–33. 41385765

[58] Zeng W Luo Y Gan D Zhang Y Deng H Liu G . Advances in Doxorubicin-based nano-drug delivery system in triple negative breast cancer. Front Bioeng Biotechnol (2023) 11:1271420. 10.3389/fbioe.2023.1271420 38047286 PMC10693343

[59] Chen G Gu X Xue J Zhang X Yu X Zhang Y Effects of neoadjuvant stereotactic body radiotherapy plus adebrelimab and chemotherapy for triple-negative breast cancer: a pilot study. Elife (2023) 12:e91737. 10.7554/eLife.91737 38131294 PMC10746137

[60] Kumar S Tailor D Dheeraj A Li W Stefan K Lee JM Uncovering therapeutic targets for macrophage-mediated T cell suppression and PD-L1 therapy sensitization. Cell Rep Med (2024) 5(9):101698. 10.1016/j.xcrm.2024.101698 39181134 PMC11524979

[61] Schmid P Salgado R Park YH Muñoz-Couselo E Kim SB Sohn J Pembrolizumab plus chemotherapy as neoadjuvant treatment of high-risk, early-stage triple-negative breast cancer: results from the phase 1b open-label, multicohort KEYNOTE-173 study. Ann Oncol (2020) 31(5):569–81. 10.1016/j.annonc.2020.01.072 32278621

[62] Schmid P Cortes J Dent R McArthur H Pusztai L Kümmel S Overall survival with pembrolizumab in early-stage triple-negative breast cancer. N Engl J Med (2024) 391(21):1981–91. 10.1056/NEJMoa2409932 39282906

[63] Dent R CortéS J Pusztai L McArthur H Kümmel S Bergh J Neoadjuvant pembrolizumab plus chemotherapy/adjuvant pembrolizumab for early-stage triple-negative breast cancer: quality-of-life results from the randomized KEYNOTE-522 study. J Natl Cancer Inst (2024) 116(10):1654–63. 10.1093/jnci/djae129 38913881 PMC11461162

[64] Schmid P Cortes J Dent R Pusztai L McArthur H Kümmel S Event-free survival with pembrolizumab in early triple-negative breast cancer. N Engl J Med (2022) 386(6):556–67. 10.1056/NEJMoa2112651 35139274

[65] Xu L Peng F Luo Q Ding Y Yuan F Zheng L IRE1α silences dsRNA to prevent taxane-induced pyroptosis in triple-negative breast cancer. Cell (2024) 187(25):7248–66.e34. 10.1016/j.cell.2024.09.032 39419025 PMC11645245

[66] Rached L Laparra A Sakkal M Danlos FX Barlesi F Carbonnel F Toxicity of immunotherapy combinations with chemotherapy across tumor indications: current knowledge and practical recommendations. Cancer Treat Rev (2024) 127:102751. 10.1016/j.ctrv.2024.102751 38729086

[67] Zheng C Liu Y Wang X Bi Z Qiu P Qiao G Clinical efficacy and biomarker analysis of neoadjuvant camrelizumab plus chemotherapy for early-stage triple-negative breast cancer: a experimental single-arm phase II clinical trial pilot study. Int J Surg (2024) 110(3):1527–36. 10.1097/JS9.0000000000001011 38116673 PMC10942181

[68] Wu S Ge A Deng X Liu L Wang Y . Evolving immunotherapeutic solutions for triple-negative breast carcinoma. Cancer Treat Rev (2024) 130:102817. 10.1016/j.ctrv.2024.102817 39154410

[69] Hunia J Gawalski K Szredzka A Suskiewicz MJ Nowis D . The potential of PARP inhibitors in targeted cancer therapy and immunotherapy. Front Mol Biosci (2022) 9:1073797. 10.3389/fmolb.2022.1073797 36533080 PMC9751342

[70] Hopkins JL Lan L Zou L . DNA repair defects in cancer and therapeutic opportunities. Genes Dev (2022) 36(5-6):278–93. 10.1101/gad.349431.122 35318271 PMC8973847

[71] Ghonim MA Ibba SV Tarhuni AF Errami Y Luu HH Dean MJ Targeting PARP-1 with metronomic therapy modulates MDSC suppressive function and enhances anti-PD-1 immunotherapy in colon cancer. J Immunother Cancer (2021) 9(1):e001643. 10.1136/jitc-2020-001643 33495297 PMC7839867

[72] Luo L Keyomarsi K . PARP inhibitors as single agents and in combination therapy: the most promising treatment strategies in clinical trials for BRCA-mutant ovarian and triple-negative breast cancers. Expert Opin Investig Drugs (2022) 31(6):607–31. 10.1080/13543784.2022.2067527 35435784 PMC9296104

[73] Teo ZL O'Connor MJ Versaci S Clarke KA Brown ER Percy LW Combined PARP and WEE1 inhibition triggers anti-tumor immune response in BRCA1/2 wildtype triple-negative breast cancer. NPJ Breast Cancer (2023) 9(1):68. 10.1038/s41523-023-00568-5 37582853 PMC10427618

[74] Agostinetto E Losurdo A Nader-Marta G Santoro A Punie K Barroso R Progress and pitfalls in the use of immunotherapy for patients with triple negative breast cancer. Expert Opin Investig Drugs (2022) 31(6):567–91. 10.1080/13543784.2022.2049232 35240902

[75] Desai NV Tan AR . Targeted therapies and the evolving standard of care for triple-negative and germline BRCA1/2-Mutated breast cancers in the High-Risk, early-stage setting. JCO Precis Oncol (2023) 7:e2200446. 10.1200/PO.22.00446 37163718

[76] Wen Y Ouyang D Zou Q Chen Q Luo N He H A literature review of the promising future of TROP2: a potential drug therapy target. Ann Transl Med (2022) 10(24):1403. 10.21037/atm-22-5976 36660684 PMC9843409

[77] Bardia A Krop IE Kogawa T Juric D Tolcher AW Hamilton EP Datopotamab deruxtecan in advanced or metastatic HR+/HER2-and triple-negative breast cancer: results from the phase I TROPION-PanTumor01 study. J Clin Oncol (2024) 42(19):2281–94. 10.1200/JCO.23.01909 38652877 PMC11210948

[78] Feng Y Lee J Yang L Hilton MB Morris K Seaman S Engineering CD276/B7-H3-targeted antibody-drug conjugates with enhanced cancer-eradicating capability. Cell Rep (2023) 42(12):113503. 10.1016/j.celrep.2023.113503 38019654 PMC10872261

[79] Dent RA Cescon DW Bachelot T Jung KH Shao ZM Saji S TROPION-Breast02: datopotamab deruxtecan for locally recurrent inoperable or metastatic triple-negative breast cancer. Future Oncol (2023) 19(35):2349–59. 10.2217/fon-2023-0228 37526149

[80] Spring LM Tolaney SM Fell G Bossuyt V Abelman RO Wu B Response-guided neoadjuvant sacituzumab govitecan for localized triple-negative breast cancer: results from the NeoSTAR trial. Ann Oncol (2024) 35(3):293–301. 10.1016/j.annonc.2023.11.018 38092228

[81] Tarekegn K Keskinkilic M Kristoff TJ Evans ST Kalinsky K . The role of immune checkpoint inhibition in triple negative breast cancer. Expert Rev Anticancer Ther (2023) 23(10):1095–106. 10.1080/14737140.2023.2265059 37771270

[82] Gulley JL Schlom J Barcellos-Hoff MH Wang XJ Seoane J Audhuy F Dual inhibition of TGF-β and PD-L1: a novel approach to cancer treatment. Mol Oncol (2022) 16(11):2117–34. 10.1002/1878-0261.13146 34854206 PMC9168966

[83] Loibl S Schneeweiss A Huober J Braun M Rey J Blohmer JU Neoadjuvant durvalumab improves survival in early triple-negative breast cancer independent of pathological complete response. Ann Oncol (2022) 33(11):1149–58. 10.1016/j.annonc.2022.07.1940 35961599

[84] O'Connell BC Hubbard C Zizlsperger N Fitzgerald D Kutok JL Varner J Eganelisib combined with immune checkpoint inhibitor therapy and chemotherapy in frontline metastatic triple-negative breast cancer triggers macrophage reprogramming, immune activation and extracellular matrix reorganization in the tumor microenvironment. J Immunother Cancer (2024) 12(8):e009160. 10.1136/jitc-2024-009160 39214650 PMC11367338

[85] Maiorano BA Lorusso D Maiorano MFP Ciardiello D Parrella P Petracca A The interplay between PARP inhibitors and immunotherapy in ovarian cancer: the rationale behind a new combination therapy. Int J Mol Sci (2022) 23(7):3871. 10.3390/ijms23073871 35409229 PMC8998760

[86] Ding JH Xiao Y Yang F Song XQ Xu Y Ding XH Guanosine diphosphate-mannose suppresses homologous recombination repair and potentiates antitumor immunity in triple-negative breast cancer. Sci Transl Med (2024) 16(728):eadg7740. 10.1126/scitranslmed.adg7740 38170790

[87] Xiang H Tu B Feng X Chen L Huang Y . Dual inhibition of TYK2 and PD-L1 boosts immune response in triple negative breast cancer. Anticancer Drugs (2025) 36(4):280–9. 10.1097/CAD.0000000000001685 39774369 PMC11884794

[88] Doroshow DB Bhalla S Beasley MB Sholl LM Kerr KM Gnjatic S PD-L1 as a biomarker of response to immune-checkpoint inhibitors. Nat Rev Clin Oncol (2021) 18(6):345–62. 10.1038/s41571-021-00473-5 33580222

[89] Rizzo A Ricci AD . Biomarkers for breast cancer immunotherapy: PD-L1, TILs, and beyond. Expert Opin Investig Drugs (2022) 31(6):549–55. 10.1080/13543784.2022.2008354 34793275

[90] Bianchini G De Angelis C Licata L Gianni L . Treatment landscape of triple-negative breast cancer - expanded options, evolving needs. Nat Rev Clin Oncol (2022) 19(2):91–113. 10.1038/s41571-021-00565-2 34754128

[91] Emens LA Loi S . Immunotherapy approaches for breast cancer patients in 2023. Cold Spring Harb Perspect Med (2023) 13(4):a041332. 10.1101/cshperspect.a041332 37011999 PMC10071416

[92] Rayson VC Harris MA Savas P Hun ML Virassamy B Salgado R The anti-cancer immune response in breast cancer: current and emerging biomarkers and treatments. Trends Cancer (2024) 10(6):490–506. 10.1016/j.trecan.2024.02.008 38521654

[93] Sood R Kumar S Laroiya I Khare S Das A Singh G Assessment of PD-L1 expression and tumor-infiltrating lymphocytes (TILs) across molecular subtypes of triple-negative breast cancer. Breast J (2020) 26(12):2424–7. 10.1111/tbj.14110 33314356

[94] Zimmerli D Brambillasca CS Talens F Bhin J Linstra R Romanens L MYC promotes immune-suppression in triple-negative breast cancer via inhibition of interferon signaling. Nat Commun (2022) 13(1):6579. 10.1038/s41467-022-34000-6 36323660 PMC9630413

[95] Michaels E Chen N Nanda R . The role of immunotherapy in triple-negative breast cancer (TNBC). Clin Breast Cancer (2024) 24(4):263–70. 10.1016/j.clbc.2024.03.001 38582617

[96] Wu SY Xu Y Chen L Fan L Ma XY Zhao S Combined angiogenesis and PD-1 inhibition for immunomodulatory TNBC: concept exploration and biomarker analysis in the FUTURE-C-Plus trial. Mol Cancer (2022) 21(1):84. 10.1186/s12943-022-01536-6 35337339 PMC8951705

[97] Manoochehri M Borhani N GerhäUSER C Assenov Y Schönung M Hielscher T DNA methylation biomarkers for noninvasive detection of triple-negative breast cancer using liquid biopsy. Int J Cancer (2023) 152(5):1025–35. 10.1002/ijc.34337 36305646

[98] Banerjee R Maitra I Bhattacharya T Banerjee M Ramanathan G Rayala SK Next-generation biomarkers for prognostic and potential therapeutic enhancement in triple negative breast cancer. Crit Rev Oncol Hematol (2024) 201:104417. 10.1016/j.critrevonc.2024.104417 38901639

[99] Gupta I Rizeq B Vranic S Moustafa AEA Al Farsi H . Circulating miRNAs in HER2-Positive and triple negative breast cancers: potential biomarkers and therapeutic targets. Int J Mol Sci (2020) 21(18):6750. 10.3390/ijms21186750 32942528 PMC7554858

[100] Adams CM Mitra R Xiao Y Michener P Palazzo J Chao A Targeted MDM2 degradation reveals a new vulnerability for p53-Inactivated triple-negative breast cancer. Cancer Discov (2023) 13(5):1210–29. 10.1158/2159-8290.CD-22-1131 36734633 PMC10164114

[101] Kumari N Jyotishi C Prajapati S Gupta R . Challenges and strategies to enhance drug efficacy of the approved drugs for chemoresistant triple-negative breast cancer: a narrative review. Trends Sci (2025) 22(4):9490. 10.48048/tis.2025.9490

[102] Shastry M Jacob S Rugo HS Hamilton E . Antibody-drug conjugates targeting TROP-2: clinical development in metastatic breast cancer. Breast (2022) 66:169–77. 10.1016/j.breast.2022.10.007 36302269 PMC9614644

[103] Leon-Ferre RA Goetz MP . Advances in systemic therapies for triple negative breast cancer. Bmj (2023) 381:e071674. 10.1136/bmj-2022-071674 37253507

[104] Corti C Venetis K Sajjadi E Zattoni L Curigliano G Fusco N . CAR-T cell therapy for triple-negative breast cancer and other solid tumors: preclinical and clinical progress. Expert Opin Investig Drugs (2022) 31(6):593–605. 10.1080/13543784.2022.2054326 35311430

[105] Liu Y Hu Y Xue J Li J Yi J Bu J Advances in immunotherapy for triple-negative breast cancer. Mol Cancer (2023) 22(1):145. 10.1186/s12943-023-01850-7 37660039 PMC10474743

